# Thoracoscopy resection of a giant solitary fibrous tumor with double pedicles and double blood supply: a case report

**DOI:** 10.1186/s12957-019-1629-1

**Published:** 2019-05-23

**Authors:** Yi Shen, Tao He, Ping Lu, Guobin Feng, Jun Zhu, Xiangan Wang

**Affiliations:** 1 Department of Thoracic Surgery, The second Affiliated Hospital of Chengdu Medical College, Nuclear Industry 416 Hospital, Chengdu, 610057 China; 2 Department of Pathology, The second Affiliated Hospital of Chengdu Medical College, Nuclear Industry 416 Hospital, Chengdu, 610057 China

**Keywords:** Solitary fibrous tumor, Double pedicles, VATS, Pleura

## Abstract

**Background:**

Solitary fibrous tumors are rare tumors derived from the pleura. A tumor generally has only one pedicle. Video-assisted thoracoscopic surgery is generally used when a tumor is small (< 10 cm), and traditional open surgery is often used when a tumor is large.

**Case presentation:**

We report a 49-year-old male patient with a space-occupying lesion in the right chest. Three-dimensional reconstruction showed that the blood supply to the tumor originated from the right lower pulmonary artery and vein. The patient was treated with minimally invasive surgery. Intraoperative exploration revealed that the tumor had two tumor pedicles, and each pedicle has an independent blood supply. The special bagging and extraction of the specimen were applied. The size of the specimen was 18 × 12 × 6 cm. Postoperative pathological examination revealed a solitary fibrous tumor.

**Conclusions:**

The solitary fibrous tumor with double pedicles and double blood supply is very rare, and it has not been reported before. Preoperative three-dimensional reconstruction plays an important role in understanding the blood supply to the tumor and the location of the tumor pedicles. After careful and comprehensive evaluation, endoscopic surgery can also be applied to the treatment of the larger fibroma (> 10 cm). The larger specimen can be extracted from the smaller incision by the “pulling carrot” method.

## Background

Solitary fibrous tumors are stromal tumors originating from dendritic stromal cells and are relatively rare in clinical practice. Solitary fibrous tumors in the chest often originate from the visceral pleura. Patients usually have no obvious symptoms, and tumors are typically identified on chest radiography upon physical examination [1], accounting for 5% of all pleural tumors [2]. A tumor generally has only one pedicle, which is supplied by pulmonary blood vessels; it often develops slowly and exhibits exogenous growth, causing no obvious symptoms. Video-assisted thoracoscopic surgery (VATS) is generally used when a tumor is small, and traditional open surgery is often used when a tumor is large. Here, we report a case of a giant solitary fibrous tumor originating from the visceral pleura with a size of approximately 18 × 12 × 6 cm. The tumor had two pedicles located in the right lower lobe. Blood was supplied by the right lower pulmonary artery and vein.

## Case presentation

**Fig. 1:**
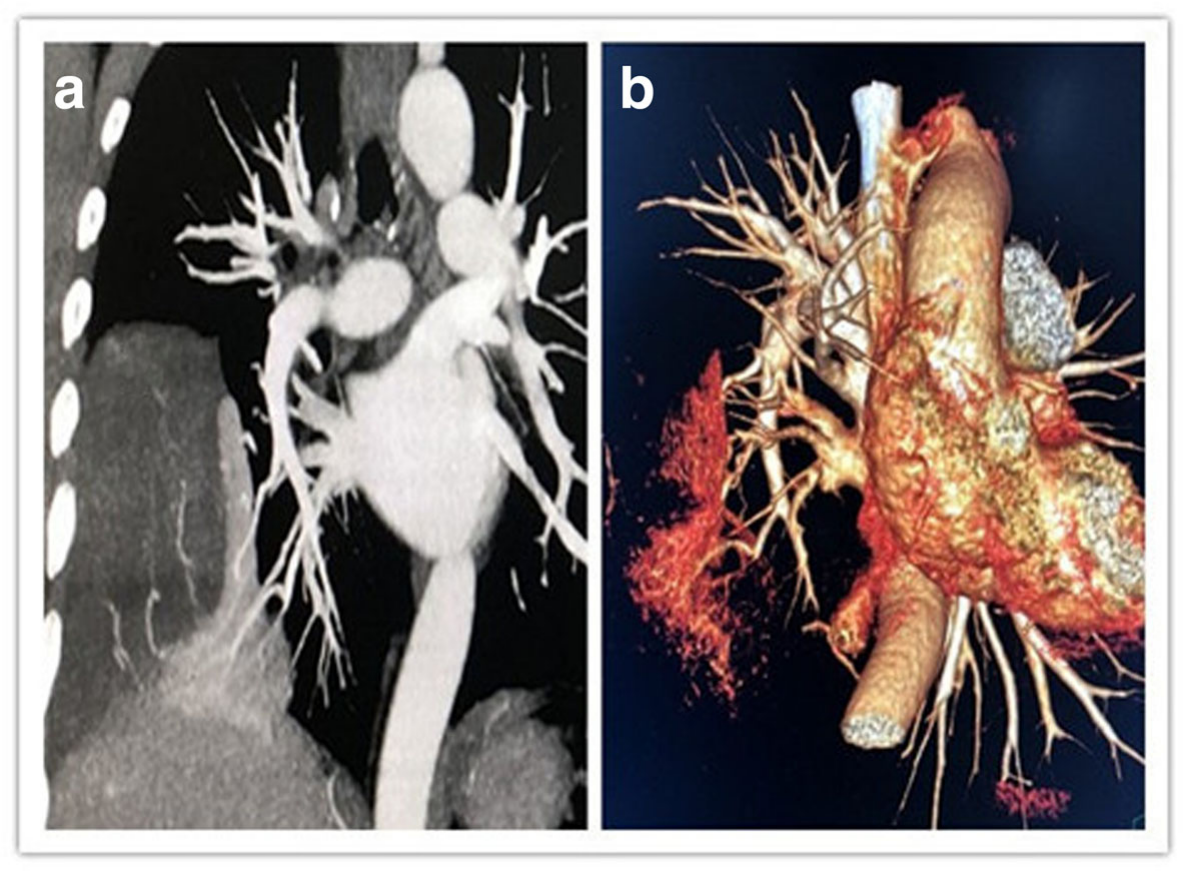
Tumor location and blood supply from the three-dimensional reconstruction. **a** The lesion is located in the right lower chest. **b** Its blood supply originated from the arteries and veins of the right lower lobe

**Fig. 2:**
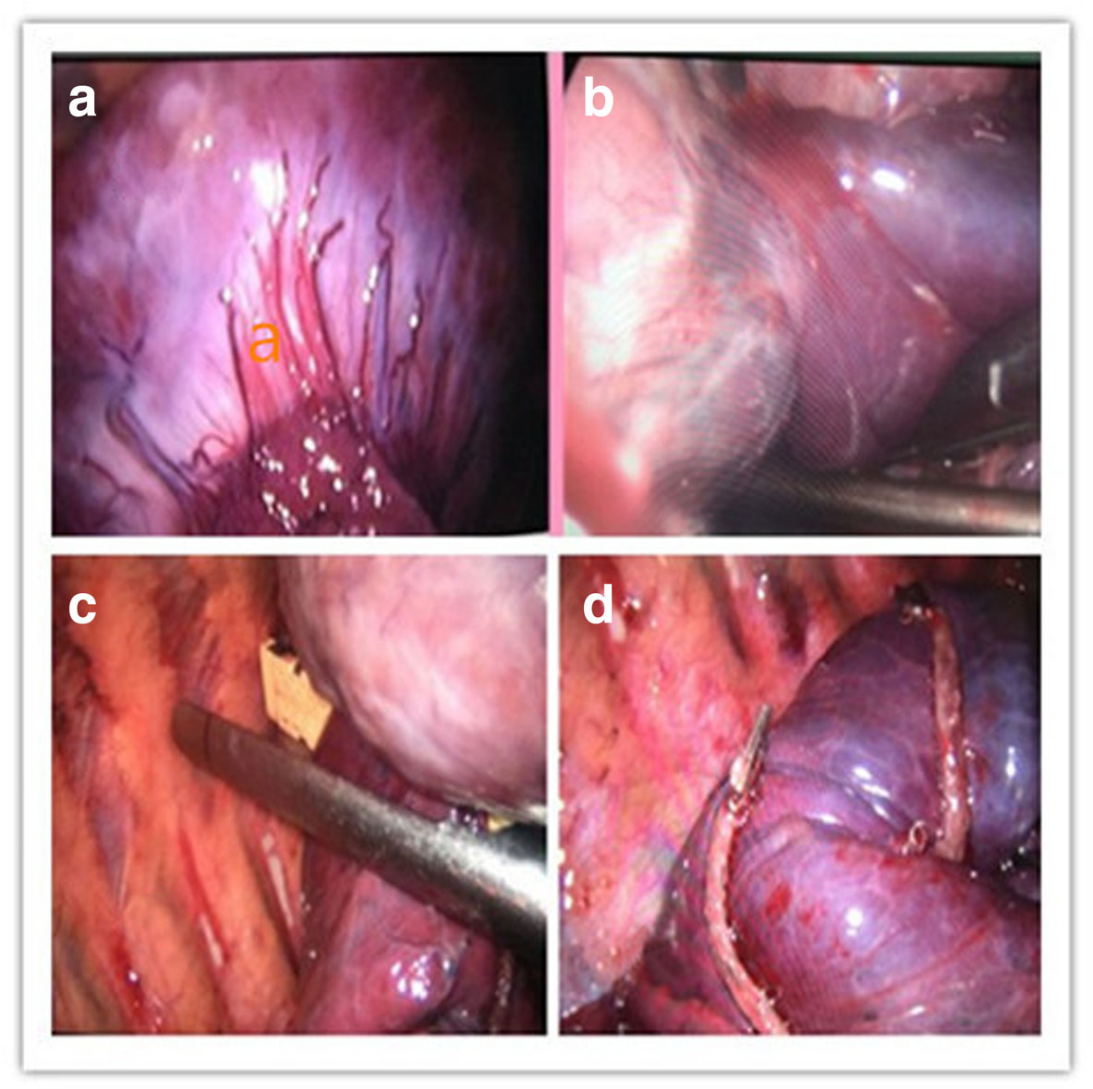
Thoracoscopic process of tumor resection. **a** Tumor and the first tumor pedicle. **b** The second tumor pedicle. **c** Resection of the tumor using a straight-line cutting stapler. **d** Two stumps after tumor resection

**Fig. 3:**
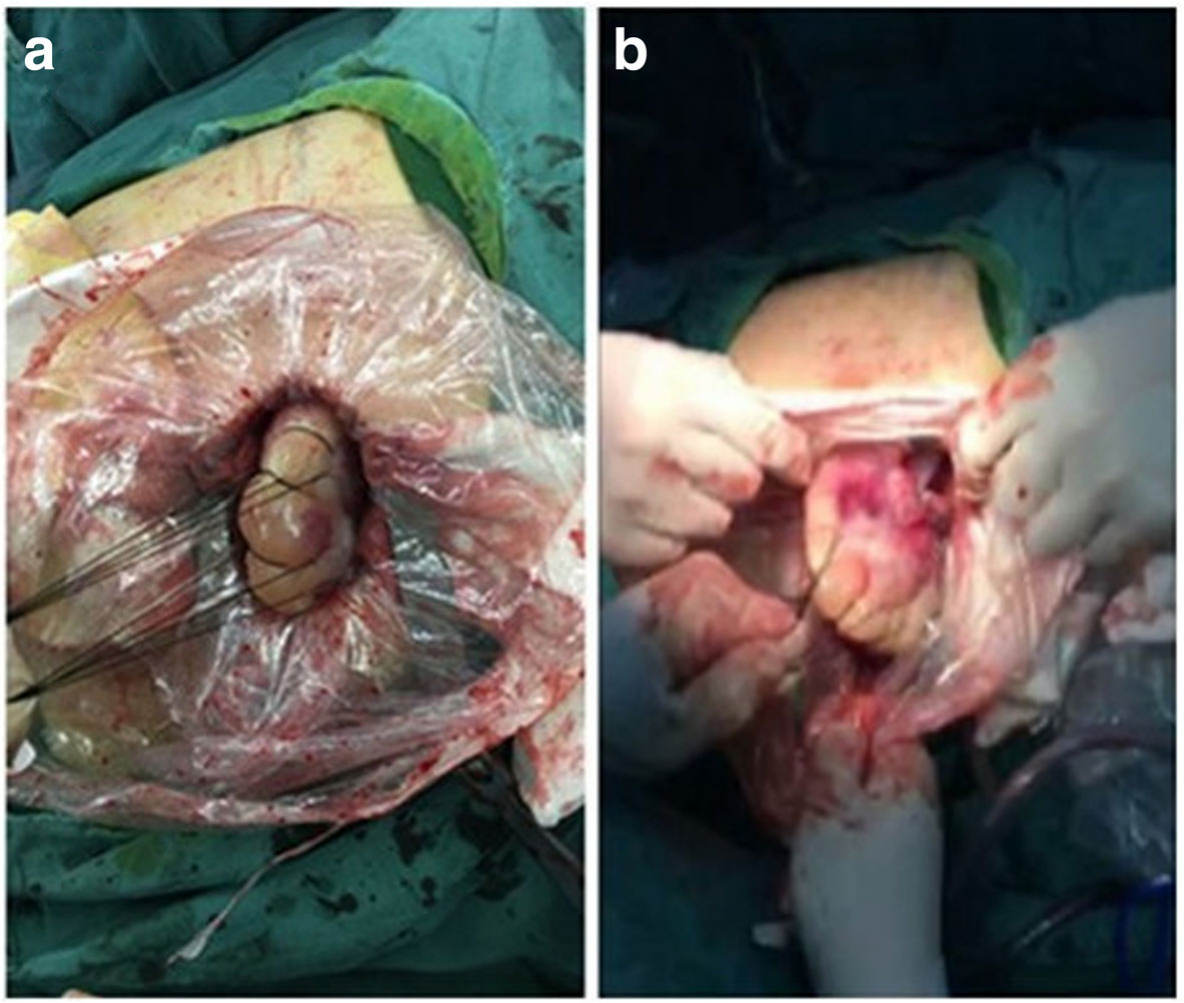
Specimen removal process. **a** Several drawstrings were intermittently sewn at the smaller end of the specimen. **b** Two people synchronously pulled the drawstrings and the specimen bag to remove the specimen

**Fig. 4:**
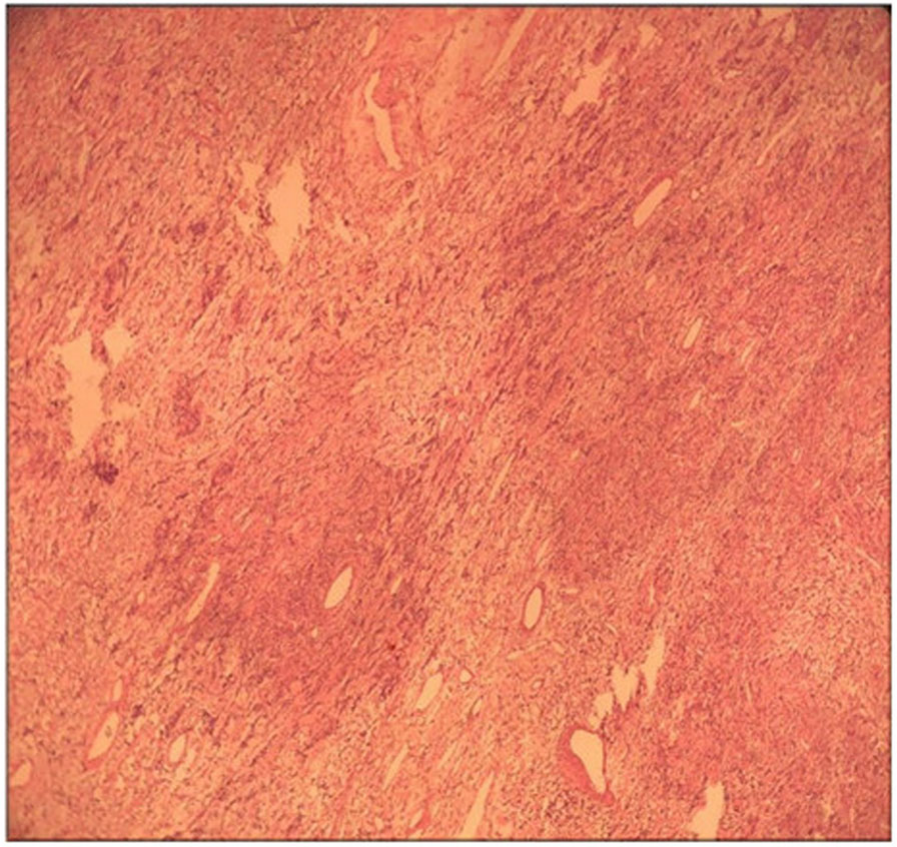
Microscopic characteristics of tumor cell. The tumor cells are slender, and the cell-rich areas and sparse areas appear alternately. The cells have no obvious atypia, and the mitotic phase is rare

A 49-year-old male patient sought treatment due to “repeated cough and sputum for one year and aggravation with chest tightness for one week.” Chest computed tomography (CT) in a local hospital revealed encapsulated effusion in the right thoracic cavity. Chest-enhanced CT after admission revealed a space-occupying lesion in the right chest. The pathological diagnosis according to percutaneous lung biopsy was a solitary fibrous tumor. Preoperative three-dimensional reconstruction showed that the blood supply to the tumor originated from the arteries and veins of the right lower lobe (Fig. 1). After complete preoperative preparation, the patient underwent resection of the tumor with single-operation-incision thoracoscopy. Incision selection is one cm for the endoscope port (at the midaxillary line of the seventh intercostal space) and two cm for the operation port (at the preaxillary line of the fifth intercostal space). Intraoperative exploration revealed that the tumor had two tumor pedicles (Fig. 2), and both were located in the right lower lobe. The tumor pedicles were intraoperatively separated using a linear stapling device. Because the specimen was large and the surface was smooth, bagging the specimen by the traditional method was difficult; therefore, the specimen was bagged by adjusting the operating table. The detailed procedure was as follows: First, the operating table was arranged with the head at a lower position, and the specimen bag was inserted into the thoracic cavity. Then, the operating table was adjusted such that the head was at a higher position to enable loading of the specimen into the specimen bag by gravity. Due to the large size of the specimen, extracting the specimen by the traditional method was difficult. Therefore, a special method named “pulling carrot” was applied to remove the specimen (Fig. 3). The specific procedure was as follows. (1) The operation port was extended to five cm. (2) Several drawstrings were intermittently sewn at the smaller end of the longitudinal axis of the specimen. The area of suturing should be as large as possible, and if necessary, additional sewing should be carried out during the process of specimen extraction. (3) Two people synchronously pulled the drawstrings and the specimen bag, with up and down, left and right shaking. The size of the specimen was 18 × 12 × 6 cm (Fig. 4). The surface of the specimen was smooth and the texture was soft and tough. Postoperative pathological examination revealed a solitary fibrous tumor immunohistochemistry: Vimentin (+), CD34 (+), bcl-2 (+), CD99 (+), SMA (−), S-100 (−), Desmin (−), P53 (−), CK (−), EMA (−), and Ki-67 positive rate of about 10%.

## Discussion and conclusion

In 1931, Klemperer and Rabin [3] first reported a case of a solitary fibrous tumor. To date, at least 800 cases of such tumors have been reported [4]. Solitary fibrous tumors often have one tumor pedicle, and the preferred treatment is surgical resection. In recent years, thoracoscopic surgery has been applied in the surgical treatment of solitary fibrous tumors. However, for larger tumors, thoracoscopic resection is difficult, and removing the specimen through a small laparoscopic incision is also difficult; therefore, traditional open surgery is still performed. Takahama et al. [5] and Schmid et al. [6] believed that for solitary fibrous tumors of the pleura with pedicles, thoracoscopic surgery should be the preferred procedure, but the larger tumor (< 10 cm) should be treated with open surgery. The maximum diameter of the tumors in this report was 18 cm. the tumor was completely resected with the single-operation-incision thoracoscopy, fully exploiting the features of clear visualization and amplification of the thoracic cavity. The tumors are tough, soft, and easy to shape, and although its size was substantially larger than the incision, it can be extracted by the “pulling carrot” method. According to WHO Classification of Tumours of Soft Tissue 4th ed 2013, Solitary fibrous tumor is a borderline tumor in this case, and the microscopic characteristics are as follows: the tumor cells are slender and the cell-rich areas and sparse areas appear alternately. The cells have no obvious atypia, and the mitotic phase is rare. However, malignant solitary fibrous tumor has several characteristics as follows: increased cell density, significant nuclear atypia, visible mitotic phase, hemorrhage, and necrosis.

The characteristics of these tumors are summarized as follows: (1) The tumor grows slowly, often causes no obvious symptoms, and is typically large when it is discovered. (2) Generally, the tumor has a complete capsule and a smooth surface and is not easy to clamp. (3) The capsule consists of fibrous tissue, and the texture is mostly tough. (4) The texture is soft and can be shaped to facilitate extraction of the specimen.

Based on this surgical case, our experiences are summarized as follows: (1) Thoracoscopic surgery causes minimal trauma and allows a quick recovery. After careful and comprehensive evaluation, endoscopic surgery can also be applied to the treatment of the larger fibroma (> 10 cm). (2) Solitary fibrous tumors are easily misdiagnosed as “pleural effusion.” Preoperative routine enhanced CT+3D reconstruction is helpful for determining the blood supply to a tumor and the location of the tumor pedicles. (3) The blood supply of the tumor and the number of tumor pedicles should be carefully explored during surgery, and the specimen should be extracted after ensuring complete resection to avoid bleeding caused by a pedicle tear. (4) When a large specimen in the thoracic cavity is bagged, the position of the operating table can be adjusted, which allows bagging of the specimen by gravity. (5) Using the “pulling carrot” method of specimen extraction, a larger specimen can be completely removed through a small endoscopic incision.
